# Impact of Conversational and Animation Features of a Mental Health App Virtual Agent on Depressive Symptoms and User Experience Among College Students: Randomized Controlled Trial

**DOI:** 10.2196/67381

**Published:** 2025-04-11

**Authors:** Stephanie Six, Elizabeth Schlesener, Victoria Hill, Sabarish V Babu, Kaileigh Byrne

**Affiliations:** 1 Department of Psychology, Clemson University, 418 Brackett Hall, Clemson, SC, 29634, United States, 1 8646563935; 2 Department of Human-Centered Computing, Clemson University, Clemson, SC, United States

**Keywords:** depression, mental health app, virtual agents, cognitive behavioral therapy, conversational agents, virtual agent, animations, college student, CBT, ANOVA, randomized controlled trial, depressive symptoms, mental disorder, mental illness, user experience, mHealth, digital health

## Abstract

**Background:**

Numerous mental health apps purport to alleviate depressive symptoms. Strong evidence suggests that brief cognitive behavioral therapy (bCBT)-based mental health apps can decrease depressive symptoms, yet there is limited research elucidating the specific features that may augment its therapeutic benefits. One potential design feature that may influence effectiveness and user experience is the inclusion of virtual agents that can mimic realistic, human face-to-face interactions.

**Objective:**

The goal of the current experiment was to determine the effect of conversational and animation features of a virtual agent within a bCBT-based mental health app on depressive symptoms and user experience in college students with and without depressive symptoms.

**Methods:**

College students (N=209) completed a 2-week intervention in which they engaged with a bCBT-based mental health app with a customizable therapeutic virtual agent that varied in conversational and animation features. A 2 (time: baseline vs 2-week follow-up) × 2 (conversational vs non-conversational agent) × 2 (animated vs non-animated agent) randomized controlled trial was used to assess mental health symptoms (Patient Health Questionnaire-8, Perceived Stress Scale-10, and Response Rumination Scale questionnaires) and user experience (mHealth App Usability Questionnaire, MAUQ) in college students with and without current depressive symptoms. The mental health app usability and qualitative questions regarding users’ perceptions of their therapeutic virtual agent interactions and customization process were assessed at follow-up.

**Results:**

Mixed ANOVA (analysis of variance) results demonstrated a significant decrease in symptoms of depression (*P*=.002; mean [SD]=5.5 [4.86] at follow-up vs mean [SD]=6.35 [4.71] at baseline)*,* stress (*P*=.005; mean [SD]=15.91 [7.67] at follow-up vs mean [SD]=17.02 [6.81] at baseline)*,* and rumination (*P*=.03; mean [SD]=40.42 [12.96] at follow-up vs mean [SD]=41.92 [13.61] at baseline); however, no significant effect of conversation or animation was observed. Findings also indicate a significant increase in user experience in animated conditions. This significant increase in animated conditions is also reflected in the user’s ease of use and satisfaction (*F*(1, 201)=102.60, *P*<.001)*,* system information arrangement (*F*(1, 201)=123.12, *P*<.001)*,* and usefulness of the application (*F*(1, 201)=3667.62, *P*<.001).

**Conclusions:**

The current experiment provides support for bCBT-based mental health apps featuring customizable, humanlike therapeutic virtual agents and their ability to significantly reduce negative symptomology over a brief timeframe. The app intervention reduced mental health symptoms, regardless of whether the agent included conversational or animation features, but animation features enhanced the user experience. These effects were observed in both users with and without depressive symptoms.

## Introduction

### Background

The prevalence of depressive symptoms within the United States drastically increased from 17 million to 21 million, a nearly 25% increase, from 2018 to 2020 during the COVID-19 pandemic [1] with young adults and women disproportionately affected [2]. To address depressive symptoms, mental health apps have emerged to offer assistance and therapeutic techniques to the public. Cognitive behavioral therapy (CBT)-based mental health apps represent a viable option to improve access to mental health resources [3]. A form of CBT, brief cognitive behavioral therapy (bCBT) has been suggested for depressive individuals as a means of maintaining the user’s attention while not requiring large amounts of the user’s time or energy. This form of CBT has successfully delivered therapeutic interventions in a time-efficient manner, around 4‐16 brief sessions [4,5] in both subclinical [6-8] and clinical populations [9]. Several bCBT-based apps, such as MoodMission [10], Pacifica [11], and SuperBetter [12], have demonstrated effectiveness in reducing depressive symptoms. Despite their effectiveness, it is unclear how specific app features may enhance user experience to maximize therapeutic benefits.

The use of virtual agents represents one avenue that may enhance mental health user experience, as virtual agents can be leveraged to mimic realistic human interactions and model social connection [13-15]. The term “virtual agent” refers to a noncontrollable, artificial intelligence (AI)-driven virtual entity, such as chatbots and embodied conversational agents (ECAs) designed to interact with users [16-18]. Chatbots communicate with the user through a textual or voice-based interface design but typically lack a visual embodiment [19]. ECAs are characterized by a human-like visual presence and have the capability to include both verbal or nonverbal communication behaviors [14,15]. While chatbots have demonstrated potential in numerous bCBT-based mental health apps, such as Woebot, Wysa, and Tess [20-22], ECAs offer a richer, more natural social presence [15], making them particularly suited for mental health interventions. Surprisingly, exceptionally few studies have evaluated the effectiveness of bCBT-based mental health apps with ECAs [6]. This study addresses this gap by incorporating an ECA-style virtual agent into the app design.

Given that a key component of ECA-style virtual agents is visual embodiment, the physical characteristics of a virtual agent may impact user experience. Research shows that similarity between a user’s demographics and an agent’s characteristics, such as gender and voice, fosters positive interactions by building trust and enhancing user motivation [23-26]. This aligns with the similarity-attraction effect, where users often prefer agents that mirror their own demographics, appearance, and voice [24]. In mental health contexts, such similarity has been shown to significantly increase users’ willingness to engage in support activities [25]. To leverage these benefits, the mental health app in this study includes customization options for the agent’s physical characteristics, aiming to create a greater sense of connection and comfort during interactions.

Beyond visual embodiment, 2 key features can be embedded into ECA-style virtual agents to convey the realism of human face-to-face interactions: conversational and animation features. Conversational behaviors, including lip-sync and speech, are used to replicate natural, verbal communication actions [15,27,28]. Virtual agent verbal cues that align with social norms, such as greetings, small talk, and thanking, foster trust and perceived knowledgeability [29,30], particularly when the agent uses a formal, familiar voice quality and style [31]. In addition, conversational agents can engage in turn-taking and provide feedback, mimicking the natural flow of human conversation [15]. Turn-taking allows users to feel that they are actively participating in the interaction, while feedback conveys that the agent is attentive and responsive [15]. A systematic review of mental health interventions leveraging conversational agents observed a significant reduction in psychological distress postintervention compared with baseline [32]. These findings underscore the preliminary efficacy of virtual agents with conversational features on mental health symptoms and suggests that virtual agent conversational feature may afford empathy and interactivity that mimics therapeutic dynamics [32]. However, few studies reviewed were empirical randomized controlled trials [32], and the variability in mental health symptoms limits understanding of how conversational features affect individuals with and without depressive symptoms.

On the other hand, animation supports natural communication by conveying nonverbal behaviors, such as facial expressions, co-speech gestures, body movements, and eye gaze [14,15]. Nonverbal cues, such as nodding and eye gaze indicate active listening and foster rapport [33-35], while facial expressions can convey emotional responsiveness [36]. It is critical for animations to appear natural, as overly expressive facial animations can seem unrealistic [37]. Natural animations encourage positive attributions toward virtual agents, such as greater acceptance, trust, credibility, and task appropriateness [31,38]. Natural animations also elicit stronger emotional responses and a greater sense of social presence compared with static or partially animated agents [39,40]. While natural animation cues, such as body movements and facial expressions, can enhance social presence, they are not always effective in conveying trustworthiness [31]. Factors like the user’s age, the relevance of the animation to the task, and the context (eg, interviews, learning, or commerce) influence how animation impacts perceived trust [31,41], and the effectiveness of such animation features in mental health contexts remains underexplored.

In human-human therapeutic interactions, body language, tone, and other social cues are critical to conveying empathy and can influence therapeutic outcomes in individuals with depression [42-44]. Research with chatbots [13] and ECAs [45] has demonstrated that individuals experiencing depressive symptoms report high perceived virtual agent empathy and user-agent working alliance with levels mirroring that of CBT-based human interventions. These findings suggest that virtual agents may be able to mirror human-human therapeutic interactions by encouraging users to feel understood and supported. Such characteristics may be especially critical for individuals with depression, who often experience negative perceptions of themselves, others, and their environment [46]. However, no studies have directly compared how these virtual agent features (eg, conversational vs animation) may influence mental health outcomes and user experience in users with and without depressive symptoms.

### Objectives

This study builds on previous work [5-13,20-22,45] in several ways. First, this study directly compares how virtual agent conversational and animation features influence user experience in the context of mental health apps using a randomized controlled trial design. Second, this study assesses whether either of these features within a bCBT-based mental health app can reduce symptoms of depression, stress, and rumination over 2 weeks. Third, this study compares these features in a sample of users with and without depressive symptoms, addressing gaps in understanding how conversational versus animation features uniquely contribute to mental health outcomes in this population.

The study hypotheses for the quantitative analyses are outlined below:

H1: Individuals will exhibit significantly lower symptoms of depression, stress, and rumination after 2 weeks. This reduction will be more pronounced in the conversational and animated conditions.

H2: Individuals will have a more positive user experience with the agent in the conversational and animated conditions.

In addition to these quantitative analyses, we will query participants’ rationale in designing their virtual agents in terms of gender and similarity to people they know through qualitative methods.

## Methods

### Study Overview

The goal of this 4-arm randomized controlled trial was to determine the effect of virtual agent conversational and animation features within a bCBT-based mental health app on user experience and change in depressive symptoms over a 2-week intervention period. The virtual agent conversational feature reflects dialogue-based interaction between the user and agent, and the virtual agent animation embodies dynamic body movements and facial expressions. Participants completed a baseline training and setup session along with baseline questionnaires through a face-to-face assessment; thereafter, participants completed the intervention and 2-week postintervention questionnaires remotely. In this section, we describe the design of the overall app and virtual agent with a focus on the manipulation of the conversational and animation features. We also describe the methodology and analytic approach used to evaluate these features in a sample of college students with and without depressive symptoms. We note that this study is based partially on dissertation work by lead author SS.

### Participants

Following previous research evaluating AirHeart [6], an a priori power analysis (*F* test, repeated measures ANOVA (analysis of variance), within-between interaction) was conducted for H1. The analysis aimed for 80% power to detect effects at *P*=.05 with 4 groups and 2 time points, based on previous effect sizes [47]. Results indicated a required minimum sample of 136 participants. Multimedia Appendix 1 provides detailed information. We sought to recruit ≥25% (170 minimum) over the minimum to account for attrition and data exclusions.

A total of 209 college students completed the study and were randomized to one of the 4 experimental conditions (n= 209; mean age 19.97 years*,* SD 2.19; Table 1). Participants were incentivized to participate with compensation in the form of course credit, extra credit, or a $20 Amazon gift card, depending on their choice. Participants were excluded if they were outside the 18‐30 years age range or did not have daily access to a smartphone. Data were excluded for 3 reasons: (1) the participant completed less than 2 CBT-based modules, (2) failed more than 1 attention check, or (3) did not submit the postintervention survey.

**Table 1.** Participant demographic Information by depressive group and condition (n=209).

Demographic characteristics	Values
**Gender, n**	
Male	168
Female	39
Nonbinary	3
**Race, n**	
White	168
Asian	19
Hispanic	10
Black	8
Bi-Racial	3
American Indian or Native	1
**Mental health diagnosis, n**	
Depression	60
Anxiety	59
ADHD^a^	21
PTSD^b^	7
Bipolar II	4
Eating disorder	2
Adjustment disorder	1
Trichotillomania	1
Mood disorder	1
**Nondepressive group**	
Mean (SD)PHQ-8 score	2.15 (1.34)
Mean (SD) age (years)	20.24 (2.49)
**Depressive group**	
Mean (SD) PHQ-8 score	9.29 (3.91)
Mean (SD) age (years)	19.84 (2.03)

a ADHD: attention deficit hyperactivity disorder

b PTSD: Post-Traumatic Stress Disorder

### AirHeart Mental Health App

The AirHeart mental health app was designed using Unity 2021 and contains all themes and features of a version published in previous work [6] but included new features, such as a help section, additional customization options for the virtual agent, and an additional resources section. The virtual agent was introduced to participants as their “virtual coach“ who joined them on their journey and guided them through CBT topics. Given the importance of customization features to foster user-agent similarity [24-26], users were able to customize numerous features of their agent, including facial features, body shape, and clothing. The majority of the conversational and animation feature design choices were motivated by past research describing the importance of both verbal (ie, lip sync animation and co-speech gesturing) and nonverbal (ie, head nods and backchanneling) behaviors in conveying natural social communication information [14,15]. All user-agent communication was conducted through natural dyadic verbal exchanges. Users initiated verbal input using a speech-to-text engine, the speech recognition system plugin, while audio-based agent dialogue was created using the following text-to-speech (TTS) engines: RTVoice Native for Android+ AmazonWeb Services Polly Standard for iOS. Additional technical details regarding app development and virtual agent customization, conversational feature, and animation feature development can be found in Multimedia Appendix 2.

### Experimental Conditions

The current experiment included 4 experimental conditions differing based on the presence or absence of conversational and animation features (conversational, animated; conversational, nonanimated; animated, conversational; nonconversational, nonanimated). All conditions had access to all app features (ie, CBT modules, journaling, mood tracker, agent customization, help section, and additional resources section).

The animation feature involved dynamic body movements and facial expressions exhibited by the virtual agent. The animated condition included human-like nonverbal body movements, mouth movements, and gestures in association with the information provided by the virtual agent. The nonanimated condition displayed a static, nonmoving virtual agent with a blank facial expression.

**Figure 1.**
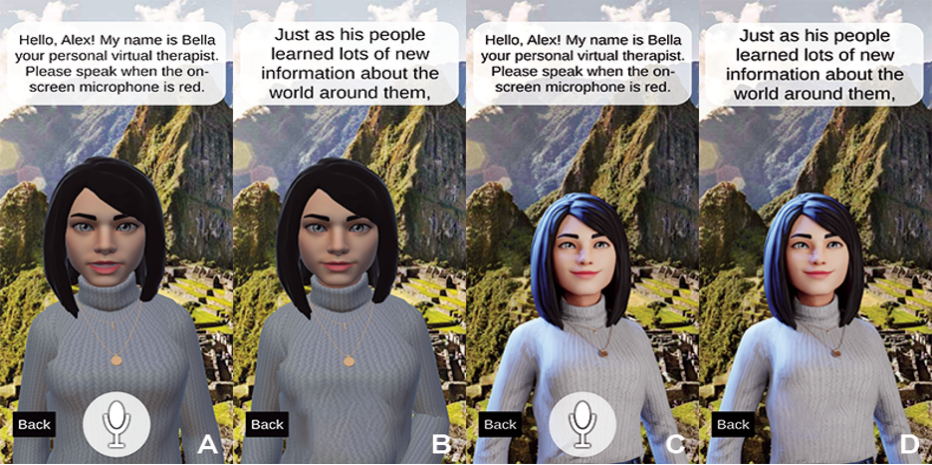
Example of a customized virtual agent in the (A) conversational, animated condition, (B) nonconversational, animated condition, (C) conversational, nonanimated condition, and (D) nonconversational, nonanimated condition. Users were able to customize their agents’ clothes, hairstyle, hair, skin, eye colors, body and face shape, facial cosmetics, and accessories. Within the conversational condition, the microphone icon provided a visual cue for users such that red indicated the microphone was on, and white indicated when it was off.

The conversational feature was characterized by user-agent interactivity in whcih question-and-response style dialogue was embedded within the CBT modules. The virtual agent asked questions or instructed the participant to complete activities aloud. A microphone icon provided a visual cue for users to engage in conversation with the agent when red, the microphone is on, and white when off. The nonconversational condition did not allow the user to add their input or respond to questions. Figure 1 shows the visualization of the virtual agent in the 4 different conditions.

### Measures

#### Depressive Symptoms Questionnaire

The Patient Health Questionnaire-8 (PHQ-8) was used to estimate depressive symptom severity over the past 2 weeks ranging from mild (0‐4) to severe (20+) [48,49].

#### Stress Symptoms Questionnaire

The Perceived Stress Scale -10 (PSS-10) is a subjective assessment of the user’s stress symptoms during the past month [50,51]. Participants’ scores ranged from 0‐40, with responses scoring <14 suggesting low stress levels and those scoring >26 suggesting high stress levels.

#### Rumination Symptoms Questionnaire

The Response Rumination Scale (RRS) is a 22-item questionnaire that measures subjective levels of rumination tendencies [52]. Responses are summed, ranging from 0‐88 with higher scores indicating more ruminative tendencies.

#### The mHealth App Usability Questionnaire

The mHealth App Usability Questionnaire (MAUQ) is a 21-item questionnaire comprised of 3 subscales: ease of use and satisfaction, system information arrangement and usefulness [53].

#### Open-Ended Qualitative Questions

Participants were asked the follow open-ended questions: (1) Did you make your virtual coach resemble yourself or someone you know? If so, why? (2) When creating your virtual coach, you were asked to select either a masculine or feminine agent. Please explain how you selected your virtual coach’s gender. What was your thought process behind the selection? (3) Do you have any suggestions for how to improve the virtual coach?

### Procedure

At the baseline assessment (Time 1), participants were first randomized to one of the 4 virtual agent conditions that varied in conversational and animation features. After providing written informed consent, they completed the mental health questionnaires (PHQ-8, PSS-10, and RRS). Users then downloaded and piloted the AirHeart app using TestFlight, a beta-testing app required for iPhones due to their additional security measures, while Android users could install the app directly. Next, they created an account, followed a tutorial to personalize their virtual agent, and completed the first CBT module. They used the app every other day for 2 weeks, which included a minimum of 8 times for full completion, but additional usage was encouraged. When participants logged into the app for the first time that day, they were prompted to complete the daily questionnaire, view their mood tracker, and then taken to the home page where they had access to the CBT modules. After the 2-week intervention, participants were contacted through email to complete postintervention questionnaires. At this assessment (Time 2), participants completed the mental health (PHQ-8, PSS-10, RRS) questionnaires again as well as the user experience questionnaire (MAU-Q) and open-ended user experience questions.

### Data Analysis

To investigate H1, separate 2 (conversational status: present vs absent) × 2 (animation status: present vs absent) × 2 (time: baseline vs postintervention symptoms) mixed effects ANOVAs was used to analyze changes in depressive, stress, and rumination symptoms, respectively. Conversational status and animation status were between-subjects factors; time was a within-subjects repeated measures factor. Sensitivity analyses were conducted that focused only on participants who reported experiencing depressive symptoms (PHQ-8 scores >4).

To assess H2 for the user experience predictions, separate 2 (conversational status: present vs absent) × 2 (animation status: present vs absent) × 2 (depressive status: depressive vs nondepressive state) multifactorial ANOVAs were performed for each of the 3 MAUQ subscales. Using the validated cutoff scores established in previous work [48,49], PHQ-8 scores ranging from 0‐4 were considered normal (or nondepressive) and scores of 5 and above were considered in a depressive state. Inclusion of this factor allowed for distinguishing whether individuals with and without current depressive symptoms had user experience preferences for the virtual agent characteristics.

For quantitative data, we conducted parametric ANOVA analyses after verifying that the data were normally distributed and error variances were equivalent [54,55]. Box’s test confirmed equality of covariance matrices, and Levene test verified homogeneity of variance. Mauchly test ensured sphericity. When appropriate, posthoc pairwise tests were conducted using the Tukey honestly significant difference for between-subjects variables and the Bonferroni adjusted alpha method for within-subjects variables. These methods are widely used in user studies and human factors research in computing [6,39,56-58]. For the open-ended qualitative questions, a reflexive thematic analysis was performed in order with the procedure specified by Braun and Clarke [59] which has been used in numerous user studies evaluating virtual agents [26,60-63]. Two researchers independently reviewed deidentified responses, manually created initial codes, and then grouped codes into categories. For each of the 3 qualitative questions, percentage agreement for categories between researchers was >85%. Researchers then reviewed the independently-generated categories, consolidated duplicates, and refined and labeled themes. Next, the study conditions (ie, conversational and animated) and depressive group (depressive vs nondepressive) were reattached to the responses to create a frequency data table.

### Ethical Considerations

The study procedures were approved by the Clemson University Institutional Review Board (IRB2021-0879) before procedures were implemented. All participants provided written informed consent before participating in the study. They were given the option to opt-out of participating. All data are deidentified. Participants were incentivized to participate with compensation in the form of course credit, extra credit, or a US $20 Amazon gift card, depending on their choice.

## Results

### Mental Health Symptoms

#### Change in Depressive Symptoms

The 2 (conversational vs nonconversational) × 2 (animated vs nonanimated) × 2 (time: baseline vs postintervention) mixed ANOVA results demonstrated a statistically significant main effect of time (*F*(1, 205)=10.06, *P*=.002; *ηp*^2^=.05), indicating that depressive symptoms were lower at 2-week follow-up (mean 5.5, SD 4.86) compared with baseline (mean 6.35, SD 4.71) across all 4 experimental conditions. There was no significant main effect of animation condition (*F*(1, 208)=.02, *P*=.91; *ηp*^2^<.001), conversational condition (*F* (1, 208)=.25, *P*=.62, *ηp*^2^=.001), nor any of the interaction effect (*P*s>.05). Multimedia Appendix 3 shows the full ANOVA results.

We note that when the 2 (conversation: present vs absent) × 2 (animation: present vs absent) × 2 (time: pre vs post) analysis is performed separately for those that meet criteria of depressive symptoms at baseline (PHQ-9 scores<6) and those that do not, the results do not differ. Thus, animation and conversation features do not significantly affect change in depressive symptoms for those with or without depressive symptoms.

#### Change in Stress Symptoms

Mixed ANOVA results showed a significant main effect of time (*F*(1, 205)=8.09, *P*=.005; *ηp*^2^=.038), such that self-reported stress levels were lower at 2-week follow-up (mean 15.91, SD 7.67) than baseline (mean 17.02, SD 6.81) across all 4 experimental conditions. The animation condition (*F*(1, 208)=.007, *P*=.93; *ηp*^2^<.001), conversational condition (*F*(1, 208)=.113, *P*=.74; *ηp*^2^=.001), and all interaction effects, (*P*s>.05) were nonsignificant (Multimedia Appendix 4).

#### Change in Rumination Symptoms

A main effect of time indicated that postintervention rumination scores were significant lower after the 2-week intervention (mean 40.42, SD 12.96) when compared with the preintervention scores (mean 41.92, SD 13.61), (*F*(1, 205)=4.88, *P*=.03; *ηp*^2^=.023) across all 4 conditions. No significant effects were ascertained for animation condition (*F*(1, 208)=.09, *P*=.76; *ηp*^2^<.001) nor the conversational condition (*F*(1, 208)=.37, *P*=.54; *ηp*^2=^.002). The interaction effect was also nonsignificant (*Ps*>.05; Multimedia Appendix 5).

### User Experience Results

#### MAUQ-Ease of Use and Satisfaction

The ANOVA analysis on MAUQ-ease of use and satisfaction scores revealed a significant main effect of animation, *F*(1, 201)=102.60, *P*<.001, *ηp*^2^=0.34. Table 2 shows the mean (SD) values on the MAUQ-ease of use and satisfaction scores, and Table 3 displays the full ANOVA results. The Tukey honestly significant difference posthoc pairwise comparisons indicated that mean MAUQ-ease of use and satisfaction scores was significantly higher when the agent was animated (mean 39.91, SD 9.51) as compared with when the agent was not (mean 23.35, SD 9.81; *P<*.001).

**Table 2.** mHealth App Usability Questionnaire scores for ease of use and satisfaction.

Variable	Score, mean (SD)
Animated, n=106	39.91 (9.51)
Nonanimated, n=103	23.35 (9.81)
Conversational, n=105	32.37 (12.53)
Nonconversational, n=104	31.12 (12.93)
Depressed, n=46	32.76 (12.82)
Not depressed, n=163	31.46 (12.71)

**Table 3.** Analysis of variance results for the mHealth App Usability Questionnaire-ease of use and satisfaction.

Effect	*F* value	*P* value	Partial Eta squared (ηp^2^)
Conversational main effect	1.23	.27	.006
Animation main effect	102.60	<.001	.34
Depressive status main effect	.86	.36	.004
Animated × conversational interaction effect	.32	.57	.002
Conversation × depressive status interaction effect	.024	.88	.0001
Animation × depressive status interaction effect	.024	.88	.0001
Conversation × animated × depressive status interaction effect	.54	.46	.003

#### MAUQ-System Information Arrangement

As presented in Table 4 and Table 5, ANOVA results for the MAUQ-system information arrangement scores showed a significant main effect of animation, *F*(1, 201)=123.12, *P*<.001, *ηp*^2^=.38. The mean MAUQ-system information arrangement scores was significantly higher (mean 30.97, SD 6.87) when the agent was animated as compared with when the agent was not (mean 17.27, SD 7.43; *P*<.001).

**Table 4.** mHealth App Usability Questionnaire scores for system information arrangement.

Variable	Score, mean (SD)
Animated, n=106	30.97 (6.87)
Nonanimated, n=103	17.27 (7.43)
Conversational, n=105	24.35 (9.74)
Nonconversational, n=104	24.09 (10.11)
Depressed, n=46	23.43 (10)
Not depressed, n=163	24.44 (9.89)

**Table 5.** Analysis of variance results for mHealth App Usability Questionnaire-system information arrangement.

Effect	*F* value	*P* value	Partial Eta squared (ηp^2^)
Conversational main effect	.16	.69	.001
Animation main effect	123.12	<.001	.38
Depressive status main effect	.44	.51	.002
Animated × conversational interaction effect	1.24	.27	.006
Conversation × depressive status interaction effect	.027	.87	.0001
Animation × depressive status interaction effect	.34	.56	.002
Conversation × animated × depressive status interaction effect	2.81	.096	.014

#### MAUQ-Usefulness

The ANOVA analysis on MAUQ-usefulness scores revealed a significant main effect of animation, revealed a significant main effect of animation, *F*(1, 201)=3667.62, *P*<.001, *ηp*^2^=.17, such that mean MAUQ-usefulness scores were significantly higher when the agent was animated (mean 32.21, SD 9.43) than when the agent was not animated (mean 22.17, SD 9.7; *P*<.001; Table 6 and Table 7).

**Table 6.** mHealth App Usability Questionnaire scores for usefulness.

Variable	Score, mean (SD)
Animated, n=106	32.21 (9.43)
Nonanimated, n=103	22.17 (9.7)
Conversational, n=105	27.81 (10.28)
Nonconversational, n=104	26.71 (11.29)
Depressed, n=46	28.59 (11.85)
Not depressed, n=163	26.89 (10.78)

**Table 7.** Analysis of variance results for the mHealth App Usability Questionnaire-usefulness.

Effect	*F* value	*P* value	Partial Eta Squared (ηp^2^)
Conversational main effect	.69	.41	.003
Animation main effect	39.91	<.001	.16
Depressive status main effect	1.40	.24	.007
Animated × conversational interaction effect	.85	.36	.004
Conversation × depressive status interaction effect	.001	.97	.000007
Animation × depressive status interaction effect	.002	.97	.000009
Conversation × animated × depressive status interaction effect	2.70	.10	.013

### Frequency Analysis for Agent Characteristic Selections

#### Agent Representativeness Selections

In total, 95 participants (45.5% of total sample) indicated that they designed the virtual agent to resemble themselves; of these participants, 55 (57.8%) were experiencing depressive symptoms than those that were not experiencing depressive symptoms (*z*=1.54, *P*=.12). Seventy-seven participants (36.8% of total sample) reported that they designed the virtual agent to resemble someone they know, such as a friend, sibling, parent, or current or former therapist. Of these participants, 40 (51.9%) reported experiencing depressive symptoms (*z*=0.33, *P*=.74). The remaining 37 participants (17.7%) reported making the virtual agent resemble a celebrity (n=3), a doctor or professional (n=2), or did not have a specific reason for their virtual agent design (n=32).

#### Agent Gender Selections

Of all participants, 84% (n=175) chose a female virtual agent, and 16% (n=34) chose a male. The majority of participants selected an agent’s gender so that it aligned with their own gender: all but 3 female participants (98.2%) chose a female virtual agent, 31 of the 39 males (79.5%) selected a male virtual agent, and both nonbinary participants chose a female agent.

### Qualitative Results

Participants were asked to explain the reason they selected the gender of their virtual agent. Responses were collected from all 209 participants, but 3 were excluded for failing to supply a usable response. Two key themes emerged: relatability (89/206, 43.2%) and trust or comfort in talking with a particular gender about one’s mental health concerns (160/206; 77.7%); note that some participants listed both reasons. Example quotes to illustrate the relatability theme are listed below:

I chose a masculine agent because I was making a model of myself.

[p#5]

Female; I am also female.

[p #116]

I chose the same gender as mine to connect better with the therapist.

[p #176]

Quotes describing the comfortability preference with a particular gender are included below:

I selected a female therapist because I feel more comfortable talking to females about my problems. This is just my personal preference.

[p #161]

I selected female because I associate women with a more nurturing nature.

[p #76]

I chose a female because my previous therapist was female and it felt more comfortable.

[p #67]

Suggestions for improving the virtual agent were collected from all 209 participants, but 39 of them failed to provide a viable answer. The 170 responses resulted in 4 different themes: (1) robotic voice or interaction, (2) lack of personalization or customization, (3) more engagement or realism, and (4) technical issues. Similar to the previous free response question, *z* score proportion tests were conducted for the depressive and nondepressive participants in each category. The robotic voice/interaction (*z*=3.36, *P*<.001) was the sole categories to reach significance. A frequency data table was created to help visualize this information (Table 8).

**Table 8.** Visualization of qualitative data: suggestions for virtual agent improvement.

Themes	Animation versus nonanimation	Conversation versus nonconversation	Examples	Depressive versus nondepressive
Robotic voice or interaction	Animated: 44/102 Nonanimated: 58/102	Conversational: 53/102 Nonconversational: 49/102	“Make it less robotic” (p. 80) “make the voice left stiff- sounds like a robot.” (p. 101) “Possibly make the voice more realistic and not as robotic” (p. 177)	Depressive: 63/102 Nondepressive: 39/102
More engagement, interaction, or connection	Animated: 7/19 Nonanimated: 12/19	Conversational: 9/19 Nonconversational: 10/19	“It didn’t really feel like we were having a conversation or that she was listening to my responses” (p. 21) “Maybe be more engaging then just talking.” (p. 59)	Depressive: 11/19 Nondepressive: 8/19
Lack of personalization	Animated: 23/41 Nonanimated: 18/41	Conversational: 27/41 Nonconversational: 14/41	“… they did not change their answers based on whether or not I responded so it did not feel very real.” (p. 83) “It seemed very scripted, and like I was just typing into a box.” (p. 150)	Depressive: 23/41 Nondepressive: 18/41
Tech or user interface issues	Animated: 8/16 Nonanimated: 8/16	Conversational: 11/16 Nonconversational: 5/16	“Map wasn’t lining up” (p. 54) “I think there should be the opportunity to rewind what the therapist says. If I missed something I would have to restart the whole module and that is frustrating.” (p. 139)	Depressive: 10/16 Nondepressive: 6/16
No suggestions	Animated: 21/38 Nonanimated: 17/38	Conversational: 14/38 Nonconversational: 24/38	“No.” (p. 30) “NA” (p. 89)	Depressive: 20/38 Nondepressive: 18/38

## Discussion

### Principal Findings

The current randomized controlled trial sought to investigate how conversational and animated components of a virtual agent within a bCBT-based mental health app might affect change in depressive symptoms and perceived user experience. Given that individuals experiencing depressive symptoms may have negative views of themselves or others and may struggle with anhedonia, low energy, amongst other symptoms [64], it is reasonable that individuals experiencing depressive symptoms may have different intervention needs or preferences compared with those who are not experiencing such symptoms. The results demonstrated that bCBT delivered through a virtual agent within a mental health app significantly reduced symptoms of depression, stress and rumination over a 2-week period, regardless of whether the agent included conversational or animation features. Consequently, these results partially support H1. The animation feature did enhance user experience, while the conversation feature had no significant impact.

While several empirically-evaluated bCBT-based mental health apps like Woebot, Wysa, Tess, and Fido [20-22,65] include virtual agents, these existing mental health apps leverage a text-based chatbot design. Such design does not allow for animation features and certain conversational feature components like natural speech dynamics and nonverbal behaviors. In addition, while Tess displays a static picture of a smiling Caucasian female in the text-based chat dialogue box [22], Woebot, Wysa, and Fido do not feature a human-like graphic and instead use animals or robots [20,21,65]. Furthermore, none of these apps feature a customizable virtual agent. In contrast, the AirHeart mental health app included a human-like, customizable virtual agent, and the conversational animated app condition featured both speech and text-based verbal capabilities, nonverbal behaviors, and dynamic animations.

Small pilot studies on virtual agent-based self-monitoring technologies have shown promise in demonstrating the feasibility and preliminary efficacy in reducing depressive symptoms [66-68]. The current study advances this work by demonstrating that virtual agent-based bCBT technology can effectively reduce depressive symptoms through a moderate-size randomized controlled trial. While conversational and animation features were expected to enhance the effectiveness of the intervention, particularly among those experiencing depressive symptoms, no added benefit of these features was observed on changes in depressive symptoms, stress, or rumination. Past work has shown that ECA-style virtual agents that mimic human-human interactions may enhance perceived empathy and working alliance with the user [45,69]. The results of the present study suggest that conversational and animation features may not be critical for establishing a meaningful connection between the virtual agent and the user in the context of bCBT mental health apps for depression. Instead, the social presence of the human-like virtual agent alone may be sufficient.

Study results indicated that users in the animated agent conditions reported higher ratings for system information arrangement (MAUQ-system information arrangement), ease of use (MAUQ-ease of use and satisfaction), and usefulness (MAUQ-usefulness) compared with those in nonanimated conditions. There was no significant difference in conversational versus nonconversational conditions; therefore, H2 is partially supported. These results suggest that animation can enhance the user experience in mental health interventions, which aligns with previous research showing that the inclusion of both nonverbal behaviors can create more human-like interactions and improve user impressions in mental health contexts [34,35]. In addition, the inclusion of such animation design has previously demonstrated a strong connection to higher levels of agent acceptance, trust, credibility, and task appropriateness [38]. These findings are crucial for developers of mental health interventions, as they underscore the importance of integrating virtual agents with natural animations, such as body, mouth, and gesture movements, to enhance user satisfaction and foster human-like interactions.

Consistent with the similarity-attraction effect [24,25], most participants (>90%) selected an agent of the same gender as themselves and designed it to resemble themselves or someone familiar, such as a friend, family member, or therapist. This preference aligns with research showing that familiarity provides comfort, particularly during vulnerability [70]. Many participants reported feeling more comfortable discussing mental health with females, citing greater relatability on emotional matters. This increased relatability may be more attributable to similarity than stereotypes of females as more emotionally aware and empathetic than men [71-73]. Indeed, while females often self-report higher empathy, a meta-analysis showed no objective gender differences in empathy [74]. The findings support research demonstrating stronger therapeutic alliances when clients and counselors share the same gender, particularly among female clients [75], and users’ preference for same-gender virtual agents [76-79]. In mental health contexts, gender synchrony has been shown to enhance trust in virtual agents, especially when paired with similar age [79]. These results highlight the importance of virtual agent gender customization for relatability in mental health app design. However, past research suggests that developers often rely on stereotypical binary gender cues, which can reinforce societal gender expectations [23]. Thus, mental health app developers and researchers should be cognizant of the limitations of stereotypical binary gender cues and enhance features that support diverse gender representation, especially in verbal and nonverbal animations.

### Limitations and Future Directions

The qualitative analysis revealed that most participants found the virtual agent’s voice robotic and suggested improvements to voice quality. It is possible that the quality of the virtual agent’s voice may have impacted the results of the conversational feature. The app used AmazonWeb Services Polly Standard Voice (iOS) and RTVoice Native (Android) for TTS, both of which can sound synthetic, similar to Siri (Apple) or Google Assistant (android). Previous research has shown that synthetic, artificial voices induce an eerie feeling [80,81], and similar results were found using a TTS agent for CBT-based emotional regulation, where participants also noted the robotic speech [82]. Future studies should explore higher quality TTS or prerecorded human voices to enhance user interactions with the virtual agent.

Furthermore, the study included pre- and 2-week postintervention measurements, but long-term follow-ups assess whether the effects of the intervention are sustained over time were not included in the study design. Additional research is needed to determine the duration of the benefits from the virtual agent-delivered bCBT mental health intervention following the conclusion of app use.

### Conclusions

This study is among the first to compare the effectiveness and user experience of a virtual agent bCBT-based mental health app in both users with and without depressive symptoms. The key findings from the study demonstrated that the app intervention was effective in reducing mental health symptoms, regardless of whether the agent included conversational or animation features, but animation features enhanced user experience. These effects were observed in both users with and without depressive symptoms. This work suggests that college students experiencing depressive symptoms may not have unique user experience requirements in mental health apps, and such findings may apply more broadly to wellness apps. The finding that virtual agent animation improves user experience in mental health apps but does not affect the intervention’s effectiveness offers valuable insight for optimizing app design, which can help guide future development of digital mental health tools that are both effective and user-friendly.

## Supplementary material

10.2196/67381 Multimedia Appendix 1 Study power analysis details.

10.2196/67381 Multimedia Appendix 2 Detailed description of the AirHeart app development, virtual agent features, and cognitive behavioral therapy modules.

10.2196/67381 Multimedia Appendix 3 Mixed analysis of variance results for change in depressive symptoms.

10.2196/67381 Multimedia Appendix 4 Mixed analysis of variance results for change in stress.

10.2196/67381 Multimedia Appendix 5 Mixed analysis of variance results for change in rumination symptoms.

10.2196/67381 Checklist 1 CONSORT checklist.

## Abbreviations

bCBT: brief cognitive behavioral therapy
CBT: cognitive behavioral therapy
ECA: embodied conversational agent
MAUQ: mHealth App Usability Questionnaire
MHealth apps: mental health applications
TTS: text-to-speech
