# Supplementary material for: Impact of Conversational and Animation Features of a Mental Health App Virtual Agent on Depressive Symptoms and User Experience Among College Students: Randomized Controlled Trial
Source: JMIR Ment Health. 2025 Apr 11;12:e67381. doi: 10.2196/67381 (PMC12007843; doi:10.2196/67381)

**Multimedia Appendix 1: Study Power Analysis Details**

To address H1, an a priori power analysis (*F*-test, repeated measures ANOVA, and within-between interaction) was performed using the G*Power 3.1 (Universität Kiel). The analysis sought to determine the number of participants necessary to maintain a power level of 80% to detect a possible effect at the *P* value of .05 level with 4 groups and 2 measurement time points. A meta-analysis was conducted to determine whether one specific app feature—gamification—improved the reduction of depressive symptoms in different mental health apps [47]. Cohen’s *f* for this experiment (*f*=0.16) was calculated from the Hedges *g* (*g*=0.32) provided in the meta-analysis, because both projects investigated mental health apps for depression. The results of this power analysis showed that a sample of 136 participants would be needed to have 80% power to detect an effect. The log for this analysis is provided below.


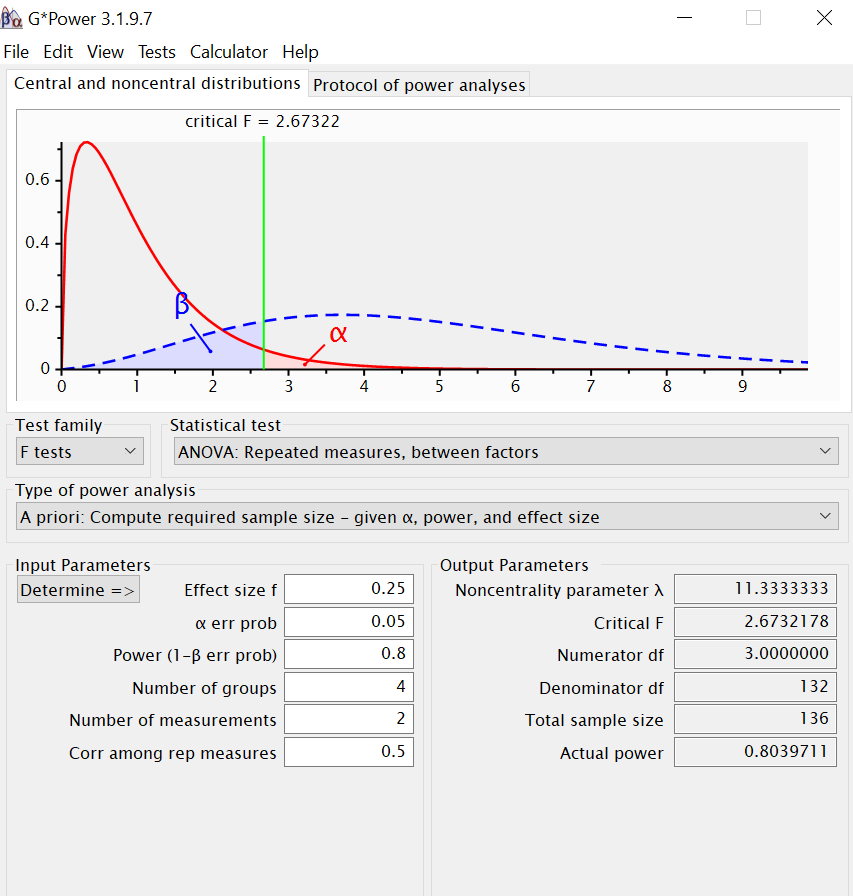

Supplement: Multimedia Appendix 1 [file mental-v12-e67381-s001.docx]
