# Supplementary material for: Impact of Conversational and Animation Features of a Mental Health App Virtual Agent on Depressive Symptoms and User Experience Among College Students: Randomized Controlled Trial
Source: JMIR Ment Health. 2025 Apr 11;12:e67381. doi: 10.2196/67381 (PMC12007843; doi:10.2196/67381)

**Multimedia Appendix 2: Detailed Description of the AirHeart App Development, Virtual Agent Features, and Cognitive Behavioral Therapy Modules**

*Development of the AirHeart Mental Health App*. The AirHeart mental health app was developed using Unity 2021. Design choices for the conversational and animation features were informed by past research on natural social communication in virtual agents [21-22]. While the AirHeart app could be placed directly on Android phones, a third-party application was necessary for downloading the app on Apple iPhones. iPhones include an extra level of security that prevents a user-created application from being directly downloaded onto the iPhone. TestFlight is a beta-testing application used to test and assess the usability of new and unreleased applications. The app can be found on the Apple App Store for free and requires 5.7 MB of storage prior to download.

*Virtual Agent Conversational Feature*. All user-agent communication was conducted via natural dyadic verbal exchanges, requiring both speech-to-text (STT) and text-to-speech (TTS) technologies. A dialogue framework utilizing the RTVoice Native (Android) + Amazon Web Services (AWS) Standard (iOS) TTS engines was enabled to provide audio-based dialogue to the participants. In general, a custom dialogue structure was developed in Unity to iterate through pre-determined lines of agent dialogue. Specific lines of dialogue were flagged as “questions”, which required user response. The dialogue system consisted of a custom dialogue object, which contained multiple lines of dialogue for the module scenario. A looping dialogue structure iterated through each line of text, which was converted into audio using the TTS engine. After the audio file finished playing, the loop continued onto the next line. This process continued until the end of the module’s dialogue object. The Speech Recognition System STT engine plug-in was used to record the participant’s response, then save the text to a local database. In the conversational condition’s dialogue object, line numbers that required user input were cued to “pause” the dialogue loop. At this point, the STT engine was turned on to record user audio. To effectively collect all user input, the dialogue loop waited three seconds after the user finished speaking before continuing to the next line of dialogue. If the participant continued speaking before the three seconds were up, the timer would stop and the STT engine would continue recording, then combine the user’s latest utterance with their previous statement. This loop would continue until the user had completed their entire response. Once the participant finished speaking, the agent would respond with one of five randomized verbal (i.e., “okay”, “I see”) and nonverbal (i.e., head nod) backchanneling responses. The flow of engines and workflows to support the conversational feature are shown in the figure below.


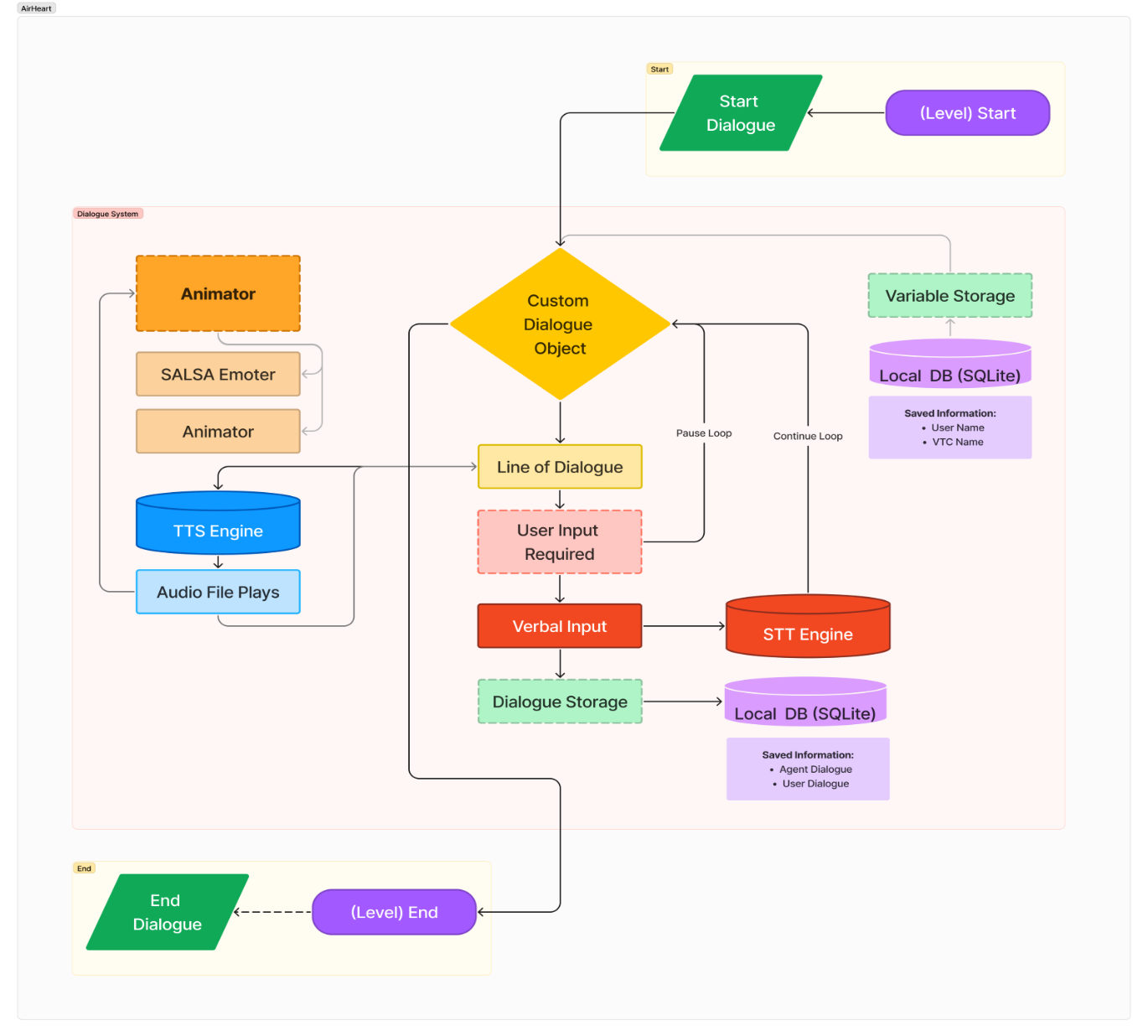


**Figure 1.** Virtual Agent Conversation Framework: At the start of a session, saved user and virtual coach names from Variable Storage are sent to the Custom Dialogue Object for personalized dialogue (e.g., “Hello Jill, my name is Bob…”). The Custom Dialogue Object generates a Line of Dialogue, which is processed by the TTS Engine and accompanied by synchronized verbal behaviors, such as co-speech gestures (via Animator) and lip-sync animations (via SALSA Emoter). After the audio playback, the system proceeds to the next dialogue line. When User Input is Required, the loop pauses and verbal input is converted to text via the STT Engine. All agent and user dialogue is stored as text in the Local Database. This loop repeats until the conversation is complete.

*Virtual Agent Animation Feature*. For the animations, the virtual agent used both verbal and non-verbal body language, specifically lip-sync movements, facial expressions, mouth movements, and body movements. All facial movement, including eye animation (i.e. blinking), lip-sync animations, and facial expressions were conducted using SALSA LipSync Suite. The virtual agent was programmed to produce two different kinds of facial expressions: positive and neutral. For example, once the user responded to an educational question (e.g. “What should Sarah do?”), the agent reacted with a head nod and positive facial expression. Neutral facial expressions were used to convey attention on the participant when they were providing personal or serious responses to questions such as, “Have you ever experienced a depressive episode?”. In line with past work showing that virtual agent nonverbal communication should be carefully designed to match the task [22], stereotypical negative facial expressions, such as frowning, was omitted in the design. Such omission served to prevent potential negative impacts on individuals with depressive symptoms who are often hyper-attuned to negative feedback [46].

Body movements, including co-speech gestural animations, waving, and head nods were created using Mixamo and Maya. Micro-expressions, such as eyebrow movements and blinking, were used randomly while talking and in idle position, to enhance the realism of the agent. The plugin SALSA LipSync Suite was attached to the virtual agent to match the visemes and phonemes with the audio, effectively providing realistic lip-sync animations in real time. Lastly, the virtual agent used randomized arm and hand movements to mimic bodily non-verbal communication in realistic conversations. This non-verbal animation included gestural animations while the agent was speaking, and head nodding to visually convey that the agent heard and understood the user’s responses.

*Virtual Agent Customization*. The virtual agent customization section utilized the online avatar creation service Ready Player Me (RPM). A RPM API was used to load avatar customization directly within the application. After completing the customization process, a web link for the RPM avatar was saved locally in a SQLite database. This link was then used by the RPM avatarLoader system to load the RPM avatar directly within the scene. RPM allowed participants access to various customization options within the application, such as clothes, hairstyles, hair, skin, eye colors, body and face shape, etc. Participants were asked to customize an agent after the initial creation of their account, but they could later change their agent’s appearance at any time afterward within the app. The virtual agent customizer section could be accessed from the map home page for convenience.

*CBT Modules.* A total of eight CBT modules were included in the MHealth app. The background displayed a realistic image of the specific location with the virtual agent in front of the current wonder of the modern world. A text box with the virtual agent’s dialogue was located above their head, at the top. This MHealth app contained the same seven bCBT modules (psychoeducation, identifying and combatting maladaptive thoughts, mindfulness and meditation, problem-solving, behavioral activation, and an overall summary) as those in our group’s previous design, with the addition of a module on episodic future thought. Episodic future thinking (EFT) involves combining prospective imagery, a part of CBT, with prompts asking about participants’ details for future enjoyable events and has been shown to increase anticipatory pleasure and joy regarding the upcoming event. These modules were originally created using traditional CBT manuals and guidance books provided to therapists and clinicians.

The eight modules took place at the seven wonders of the modern world plus an additional location featuring the students’ university. Participants were encouraged to use their new skills and techniques learned from the app during the experiment. The order of the modules can be visualized below.

**Order of CBT Modules within the MHealth App**


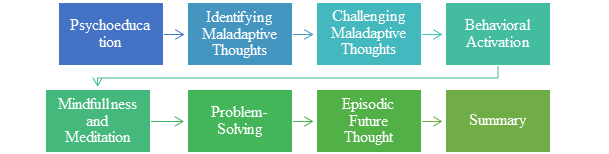

Supplement: Multimedia Appendix 2 [file mental-v12-e67381-s002.docx]
