# Supplementary material for: Impact of Conversational and Animation Features of a Mental Health App Virtual Agent on Depressive Symptoms and User Experience Among College Students: Randomized Controlled Trial
Source: JMIR Ment Health. 2025 Apr 11;12:e67381. doi: 10.2196/67381 (PMC12007843; doi:10.2196/67381)
Supplement: Multimedia Appendix 3 [file mental-v12-e67381-s003.docx]

**Multimedia Appendix 3**

Mixed ANOVA Results for Change in Depressive Symptoms

| **Means (*M*)** | | | **Standard Deviation (*SD*)** | | |
| --- | --- | --- | --- | --- | --- |
| Animated  *N* = 107 | PRE: 6.32  POST: 5.60 | | Animated | | PRE: 4.84  POST: 5.00 |
| Non-Animated  *N* = 102 | PRE: 6.38  POST: 5.40 | | Non-Animated | | PRE: 4.59  POST:  4.73 |
| Conversational  *N* = 105 | PRE: 6.49  POST: 5.67 | | Conversational | | PRE: 4.53  POST: 4.97 |
| Non-Conversational  *N* = 104 | PRE: 6.21  POST: 5.50 | | Non-Conversational | | PRE: 4.90  POST: 4.86 |
| **Effect** | | **F-Value** | | **p-value (*p*)** | **Partial Eta Squared (**ηp^2^) |
| **Time Main Effect* | | *10.60* | | *.002* | *.05* |
| Animated Main Effect | | .015 | | .91 | <.001 |
| Conversational Main Effect | | .25 | | .62 | .001 |
| Animated X Conversational Interaction Effect | | .037 | | .85 | <.001 |
| Time X Animated Interaction Effect | | .24 | | .62 | .001 |
| Time X Conversational Interaction Effect | | .02 | | .89 | < .001 |
| Time X Animated X Conversational Interaction Effect | | .76 | | .38 | .004 |

*Significant using alpha = 0.05
