# Supplementary material for: Impact of Conversational and Animation Features of a Mental Health App Virtual Agent on Depressive Symptoms and User Experience Among College Students: Randomized Controlled Trial
Source: JMIR Ment Health. 2025 Apr 11;12:e67381. doi: 10.2196/67381 (PMC12007843; doi:10.2196/67381)
Supplement: Multimedia Appendix 4 [file mental-v12-e67381-s004.docx]

**Multimedia Appendix 4**

Mixed ANOVA Results for Change in Self-Reported Stress

| **Means (*M*)** | | | **Standard Deviation (*SD*)** | | |
| --- | --- | --- | --- | --- | --- |
| Animated  *N* = 107 | PRE: 16.78  POST: 16.09 | | Animated | | PRE: 6.50  POST: 7.75 |
| Non-Animated  *N* = 102 | PRE: 17.27  POST: 15.73 | | Non-Animated | | PRE: 7.14  POST: 7.61 |
| Conversational  *N* = 105 | PRE: 16.97  POST: 15.67 | | Conversational | | PRE: 6.62  POST: 7.72 |
| Non-Conversational  *N* = 104 | PRE: 17.07  POST: 16.16 | | Non-Conversational | | PRE: 7.03  POST: 7.64 |
| **Effect** | | **F-Value** | | **p-value (*p*)** | **Partial Eta Squared (**ηp^2^) |
| **Time Main Effect* | | *8.09* | | *.005* | *.038* |
| Animated Main Effect | | .007 | | .92 | <.001 |
| Conversational Main Effect | | .11 | | .74 | .001 |
| Animated X Conversational Interaction Effect | | .38 | | .54 | .002 |
| Time X Animated Interaction Effect | | 1.20 | | .28 | .006 |
| Time X Conversational Interaction Effect | | .24 | | .62 | .001 |
| Time X Animated X Conversational Interaction Effect | | .26 | | .61 | .001 |

*Significant using alpha = 0.05

We note that when the mixed ANOVA analysis is performed separately for those that meet criteria of depressive symptoms at baseline (PHQ-9 scores <6) and those that do not, the results do not differ. Thus, animation and conversation features do not significantly affect change in stress for those with or without depressive symptoms.
