# Supplementary material for: Impact of Conversational and Animation Features of a Mental Health App Virtual Agent on Depressive Symptoms and User Experience Among College Students: Randomized Controlled Trial
Source: JMIR Ment Health. 2025 Apr 11;12:e67381. doi: 10.2196/67381 (PMC12007843; doi:10.2196/67381)
Supplement: Multimedia Appendix 5 [file mental-v12-e67381-s005.docx]

**Multimedia Appendix 5**

Mixed ANOVA Results for Change in Rumination Symptoms

| **Means (*M*)** | | | **Standard Deviation (*SD*)** | | |
| --- | --- | --- | --- | --- | --- |
| Animated  *N* = 107 | PRE: 41.48  POST: 40.33 | | Animated | | PRE: 13.25  POST: 13.19 |
| Non-Animated  *N* = 102 | PRE: 42.40  POST: 40.51 | | Non-Animated | | PRE: 14.04  POST: 12.79 |
| Conversational  *N* = 105 | PRE: 42.22  POST: 41.21 | | Conversational | | PRE: 13.51  POST: 12.34 |
| Non-Conversational  *N* = 104 | PRE: 41.63  POST: 39.63 | | Non-Conversational | | PRE: 13.78  POST: 12.97 |
| **Effect** | | **F-Value** | | **p-value (*p*)** | **Partial Eta Squared (**ηp^2^) |
| **Time Main Effect* | | *4.88* | | *.028* | *.023* |
| Animated Main Effect | | .09 | | .76 | <.001 |
| Conversational Main Effect | | .37 | | .55 | .002 |
| Animated X Conversational Interaction Effect | | .39 | | .54 | .002 |
| Time X Animated Interaction Effect | | .34 | | .56 | .002 |
| Time X Conversational Interaction Effect | | .52 | | .47 | .003 |
| Time X Animated X Conversational Interaction Effect | | 2.45 | | .12 | .012 |

*Significant using alpha = 0.05

We note that when the mixed ANOVA analysis is performed separately for those that meet criteria of depressive symptoms at baseline (PHQ-9 scores <6) and those that do not, the results do not differ. Thus, animation and conversation features do not significantly affect change in rumination for those with or without depressive symptoms.
